# Mobile apps in dermatology: Bridging innovation and care

**DOI:** 10.1111/srt.13640

**Published:** 2024-02-29

**Authors:** Simona A. Alomary, Elyse Mackenzie, Samavia Khan, Babar K. Rao

**Affiliations:** ^1^ Department of Dermatology Rutgers New Jersey Medical School Newark New Jersey USA; ^2^ Department of Dermatology Rutgers Robert Wood Johnson Medical School Piscataway New Jersey USA

## 1 INTRODUCTION

Dermatological drug development has experienced a booming influx over the past decade, providing novel solutions for a diverse array of skin conditions.^1^ This growth is attributed to both advancements in biological therapeutics and a heightened understanding of treatment options amongst providers and patients. While the advent of novel pharmacological targets holds potential for improved patient outcomes, dermatologists face the challenge of staying abreast of drug developments while delivering effective clinical care.^2^ In response, mobile applications have emerged in hopes of bridging the gap between innovations and clinical practice.^3^ While over 50 000 mobile health (mHealth) apps were available on the Apple App store in 2022, it is unclear how many of these applications can guide providers in dermatological medication management.^4^ This paper investigates the ability of top‐rated mobile iOS dermatological applications to incorporate new drug development data into their use as clinical support tools. We ultimately stress the need for development of a comprehensive drug‐centric dermatological app for the betterment of providers, patients, and the wider healthcare system.

## 2 METHODS

A recent US‐based review identified a total of 632 mobile health (mHealth) applications on Android and Apple devices that are specific to dermatology.^1^ The review further categorized applications by intended use. The majority of identified applications fell into the category of “disease guide,” with 163 apps (25.8%) focusing on including etiology, pathogenesis, and treatment of specific skin diseases. Other categories included “self‐surveillance/diagnosis,” “teledermatology,” “general dermatology reference,” “educational aid,” and more. Of these applications, 395 (62.5%) were marketed towards patients, 203 (32.1%) were marketed towards providers, and 34 (5.4%) were marketed towards both.

Authors Oullette and Rao summarize characteristics of top dermatological apps based on the number of reviews per category. However, this summary does not specify whether these apps provide dermatological drug‐specific guidance for healthcare professionals. We investigated whether the top provider‐facing iOS applications in each category had the capability to provide updated information about new drugs, treatment guidelines, and/or drug‐related research findings (Table 1). It is worth mentioning that Oullette and Rao specifically focused on analyzing the top‐reviewed apps in each category, with the intention of capturing the mobile tools most frequently utilized by providers. Future studies could consider conducting a more comprehensive scoping review of all 632 dermatological apps that are currently available.

**TABLE 1:** Top provider‐facing iOS applications and their drug‐specific capabilities.

Category	Name	No. of Reviews	Platform	Target audience	Drug‐specific capabilities?
General dermatology Reference	**VisualDx**	**564**	**Apple, android**	**Providers**	**Yes**
	Skin disease treatment symptoms and diagnosis 2019	123	Apple, android	Patients, providers	N/A^a^
Educational aid	Dermatology by Dr. Manish Soni	161	Apple, android	Providers	N/A^b^
	Dermoscopy two step algorithm	128	Apple, android	Providers	No
	**iDoc Academy**	**113**	**Apple, android**	**Providers**	**Yes**
	**Top Derm: A game for dermatologists**	**73**	**Apple, android**	**Providers**	**Yes**
Conference	AAD meetings	22	Apple, android	Providers	No
	Dermacon 2020	12	Apple, android	Providers	No
	Fall clinical	3	Apple, android	Providers	No
Calculator	Melanoma test	13	Apple, android	Providers	No
	Skin lymphoma	8	Apple	Providers	No
Photography	**HumazeMD**	**8**	**Apple, android**	**Providers**	**Yes**
Research	PeDRA research	24	Apple, android	Providers	No

^a^ No longer available on app store.

^b^ Unavailable in United States.

## 3 RESULTS

Of the 632 applications identified in the previous study, only 17 (2.68%) were provider‐facing, placed within the top five most reviewed applications for each category, and available on iOS.^1^ Of these 17 applications, we identified 4 that included information about treatment options for dermatological conditions: “VisualDx”, “iDoc Academy”, “TopDerm”, and “HumazeMD”. This accounts for <1% of all identified applications. Inclusion criteria included apps containing one or more condition‐specific treatment options, one or more drug‐specific interaction, one or more scholarly citation regarding a suggested treatment, and/or general education information about one or more dermatological treatments. A summary of the capabilities for the four identified apps are listed below. Notably, all of the listed applications in Table 1 are free to download.

### 3.1 VisualDx

“VisualDx”, categorized as a general dermatology reference, is a clinical decision support system designed to enhance medical decisions, aid therapeutic decisions, and improve patient safety.^1^ Relevant features include evidence‐based treatment options for dermatological conditions and a smart search for drug reactions. It also provides healthcare professionals access to an extensive repository of medical imagery, peer‐reviewed content, handouts and tools for fostering patient engagement, and webinars.

### 3.2 iDoc Academy

“iDoc Academy”, categorized as an educational aid, is an e‐learning platform for dermatologists and cosmetologists.^1^ The main feature of this application is the availability of virtual courses, which detail treatment methods for specific conditions (i.e., “Acne Scar Revision—Comparison of Surgical and Non‐Surgical Treatments”) through a structured interface. By collating information into detailed courses, it offers providers the ability to acquire valuable knowledge in an organized and efficient manner.

### 3.3 Top Derm

“Top Derm”, categorized as an educational aid, contains interactive medical video games covering a variety of dermatology topics.^1^ It includes treatment‐related games (i.e., “Intervention Selection”) that are embedded with citations, which become available once a correct answer is selected. “Top Derm” also incorporates advanced DeepSkinFX Generator technology, which allows users to view ultra‐high‐resolution depictions of various skin disorders on anybody region and across all skin tones. This innovation, paired with collections like “Intervention Selection”, helps create an engaging and interactive learning environment for providers.

### 3.4 HumazeMD

“HumazeMD”, categorized as a research application, provides a platform for clinicians to organize and showcase before and after photos from patient procedures.^1^ Relevant features include the ability to tag each submission by treatment and view other providers’ treatment methods, with the ultimate goal of discovering better patient outcomes. HumazeMD differs from the other applications in that it offers a community‐based approach to patient care through the collaboration that comes with sharing these before and after photos. This gives clinicians the ability to analyze different patient outcomes after specific treatments, allowing them to share knowledge and learn from one another.

## 4 CONCLUSIONS

Among the 632 mobile applications analyzed, less than 1% both met our criteria and also encompassed elements pertinent to drug development. Given both the scarcity of dermatological apps focused on drug development and the limited offerings of the existing ones, there is a demonstrated need for a more comprehensive dermatological application. Such an app could provide the latest drug development information, treatment guidelines, and educational tools for effective patient care and physician education. Additionally, this application could include patient‐specific features, such as medication reminders and symptom trackers. This would create a more streamlined approach, allowing more time for direct patient care and fostering more informed healthcare decisions for providers.

## CONFLICT OF INTEREST STATEMENT

The authors have no conflict of interest to report.

## Data Availability

Data sharing not applicable to this article as no datasets were generated or analyzed during the current study.
